# A bibliometric analysis of inflammation and thyroid carcinoma: research trends and future perspectives

**DOI:** 10.3389/fonc.2025.1593006

**Published:** 2025-08-01

**Authors:** Yanhui Feng, Caiqi Huang, Xiaoman Liu, Zhesi Xiao, Lu Wang, Zhengjia Lu, Jia Ming

**Affiliations:** Department of Breast and Thyroid Surgery, The Second Affiliated Hospital of Chongqing Medical University, Chongqing, China

**Keywords:** thyroid carcinoma, inflammation, bibliometric analysis, Web of Science, VOSviewer, CiteSpace

## Abstract

**Background and objectives:**

Thyroid carcinoma, one of the most prevalent endocrine malignancies, has witnessed a gradual increase in incidence in recent years. Accumulating evidence has demonstrated that inflammation plays a pivotal role in the pathogenesis, progression, and prognosis of thyroid carcinoma, with a significant association between certain malignancies and chronic inflammatory processes. This study conducts a bibliometric analysis of literature on inflammation and thyroid carcinoma over 24 years, aiming to identify trends and research dynamics. The findings are expected to deepen understanding of thyroid carcinoma mechanisms and guide new therapeutic strategies.

**Methods:**

We used the advanced search function of the Web of Science Core Collection to systematically screen and curate articles on inflammation and thyroid carcinoma published between 2000 and 2024. Using Microsoft Excel 2019, we analyzed and visualized publication volume and growth trends. For co-occurrence and clustering analysis of countries, institutions, authors, journals, references, and keywords, we employed VOSviewer, CiteSpace, and the ‘bibliometrix’ package in R. Keyword visualization identified 10 major clusters, including sodium iodide symporter, total thyroidectomy, peroxisome proliferator-activated receptor, papillary thyroid microcarcinoma, therapeutic radiopharmaceuticals, zoological gardens, endoscopic thyroidectomy, mixed cryoglobulinemia, fine needle aspiration, and clinical evaluation. The most frequent keywords were cancer, thyroid cancer, and inflammation.

**Results:**

We included a total of 1,441 articles published between 2000 and 2024, contributed by 8,326 authors from 2,054 institutions across 70 countries. These articles were published in 625 journals, encompassing 59,808 references and 6,340 keywords. The publication output demonstrated a consistent upward trend over the study period. Among the contributing nations, China emerged as the most prolific country in terms of publication volume. The leading institution was the University of Pisa in Italy. The most productive author was Antonelli, Alessandro, and the leading journal was *Thyroid*.

**Conclusion:**

This bibliometric analysis shows that research on inflammation and thyroid carcinoma is a rapidly evolving field, marked by diverse themes and in-depth studies. Advances in technology and extensive research are expected to clarify how inflammation drives thyroid carcinoma initiation and progression. This deeper understanding will lead to new approaches in diagnosis, prevention, and treatment of thyroid carcinoma.

## 1 Introduction

Thyroid carcinoma is the most common endocrine-related malignancy, accounting for 3% of global cancer incidence (1). According to Global Cancer Statistics 2022, there were 821,173 new cases and 47,485 deaths from thyroid cancer in 2022. Thyroid cancer has become a significant global health issue, with its incidence steadily increasing over the past few decades, profoundly impacting patients’ physical health and quality of life (2, 3). Thyroid cancer originates from thyroid follicular cells and parafollicular cells. Among these, three main malignant types exist: well-differentiated thyroid carcinoma (WDTC), poorly differentiated thyroid carcinoma (PDTC), and anaplastic thyroid carcinoma (ATC). WDTC is further divided into papillary thyroid carcinoma (PTC) and follicular thyroid carcinoma (FTC). These two types exhibit distinct histological features and retain key diagnostic markers, which are crucial for diagnosis and treatment strategies (4). PDTC primarily includes insular thyroid carcinoma (ITC) and large-cell PDTC. The rise in thyroid carcinoma incidence is most pronounced in Asia, particularly in South Korea (5). Although some understanding of thyroid tumor etiology exists, its exact causes remain incompletely understood. Radiation exposure and iodine deficiency or excess are widely recognized as key risk factors. Additionally, dietary habits and living environments are considered significant contributors. These factors collectively play an important role in the development of thyroid tumors (6).

Inflammation is a complex biological defense mechanism aimed at protecting the body from harmful substances and is widely recognized as one of the seven hallmarks of cancer. Over the past few decades, the role of inflammation in thyroid carcinoma has been actively studied. The causal link between inflammation and cancer was first established by Rudolf Virchow in 1863, who observed leukocyte infiltration in tumor tissues, providing direct evidence of their profound biological connection. This discovery deepened our understanding of cancer mechanisms and laid the theoretical foundation for subsequent cancer research and therapy (7). Evidence suggests a positive correlation between chronic inflammation and increased thyroid carcinoma risk. Inflammatory immune cells and mediators (e.g., interleukins, cytokines) influence thyroid carcinoma development. These cells and mediators enrich the tumor stroma, participating in tissue repair, remodeling, and angiogenesis (8). The cancer stroma contains both anti-tumor inflammatory cells and activated immune cells that promote tumor immune responses. The balance between anti-tumor and pro-tumor immune responses ultimately determines cancer suppression or progression (9, 10). The tumor microenvironment (TME) also plays a pivotal role in the initiation and progression of thyroid carcinoma. The active presence of lymphocytes, neutrophils, macrophages, and a range of chemokines within the TME not only directly contributes to tumor development and metastasis (11)but is also closely associated with metastatic dysregulation, tumor-related proliferation, and apoptotic processes (12). Studies show that the TME acts as a reservoir for pro-tumor and pro-angiogenic cytokines produced by tumor and immune cells, further stimulating tumor cell proliferation and immune cell recruitment, thereby advancing cancer progression (13). Additionally, multiple inflammatory factors (e.g., NF-κB, IL-6, IL-1β) interact through complex signaling networks to promote tumorigenesis and progression. These factors are not only associated with thyroid carcinoma staging and prognosis but also represent potential therapeutic targets (14). In thyroid carcinoma research, inflammation-related studies have gained significant attention. We systematically analyzed relevant articles from the past 24 years to identify core themes, trends, and future directions, providing guidance for further studies. This analysis clarifies inflammation’s role in thyroid carcinoma initiation, progression, and its impact on the TME, while exploring inflammatory factors as prognostic markers and therapeutic targets. Our findings aim to offer new strategies for prevention, diagnosis, and treatment.

Bibliometrics is a quantitative analysis tool that applies mathematics and statistics to literature research, capable of processing and analyzing large, diverse datasets. Its strengths lie in objectively and intuitively showcasing the historical activities and achievements of academic research, offering a macro perspective on the evolution of scientific knowledge. Key metrics include publication volume, citation counts, author and institutional distributions, keyword analysis, and journal impact factors, helping to minimize bias in paper evaluation (15). It focuses not only on publication volume but also delves into the quality, distribution, and impact of literature across multiple dimensions. Alan Pritchard first introduced the concept of bibliometrics in 1969, defining it as the application of mathematical and statistical methods to analyze publication counts, revealing the processes of written communication and the nature and trends of disciplinary development. Pritchard’s work marked the birth of bibliometrics as an independent discipline, aiming to understand the research landscape, trends, and frontiers of specific fields by analyzing patterns and characteristics of academic papers (16). Currently, bibliometric analysis and visualization are widely applied across various fields to analyze research status, hotspots, and trends (17).

## 2 Materials and methods

**Figure 1:**
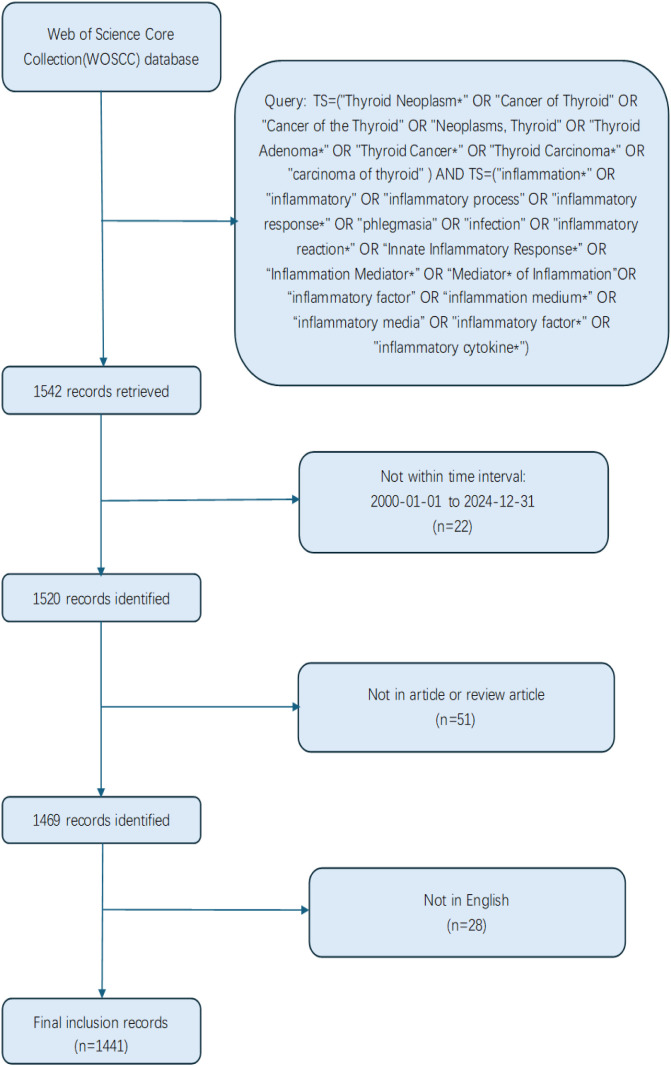
Depicts the literature screening process utilized in this study.

Web of Science (WoS, Clarivate Analytics, Philadelphia, PA, USA), encompassing over 12,000 international academic journals, is one of the most authoritative and influential data platforms globally. It features a critical citation indexing function and is extensively utilized for bibliometric analysis in the fields of medicine and health sciences (18–20). On November 12, 2024, we conducted a comprehensive literature search for this study using the advanced search strategy of the Web of Science Core Collection (WOSCC), covering research published between 1999 and 2024. The key search themes were as follows: TS=(“Thyroid Neoplasm*” OR “Cancer of Thyroid” OR “Cancer of the Thyroid” OR “Neoplasms, Thyroid” OR “Thyroid Adenoma*” OR “Thyroid Cancer*” OR “Thyroid Carcinoma*” OR “carcinoma of thyroid”) AND TS=(“inflammation*” OR “inflammatory” OR “inflammatory process” OR “inflammatory response*” OR “phlegmasia” OR “infection” OR “inflammatory reaction*” OR “Innate Inflammatory Response*” OR “Inflammation Mediator*” OR “Mediator* of Inflammation” OR “inflammatory factor” OR “inflammation medium*” OR “inflammatory media” OR “inflammatory factor*” OR “inflammatory cytokine*”). The search was restricted to English-language publications, and the document types were limited to “article” and “review article.” The publication years were set from 2000 to 2024. After final selection, a total of 1,441 articles were included for data analysis. The specific exclusion criteria are illustrated in **Figure 1**.

## 3 Results

### 3.1 Publication characteristics

**Figure 2:**
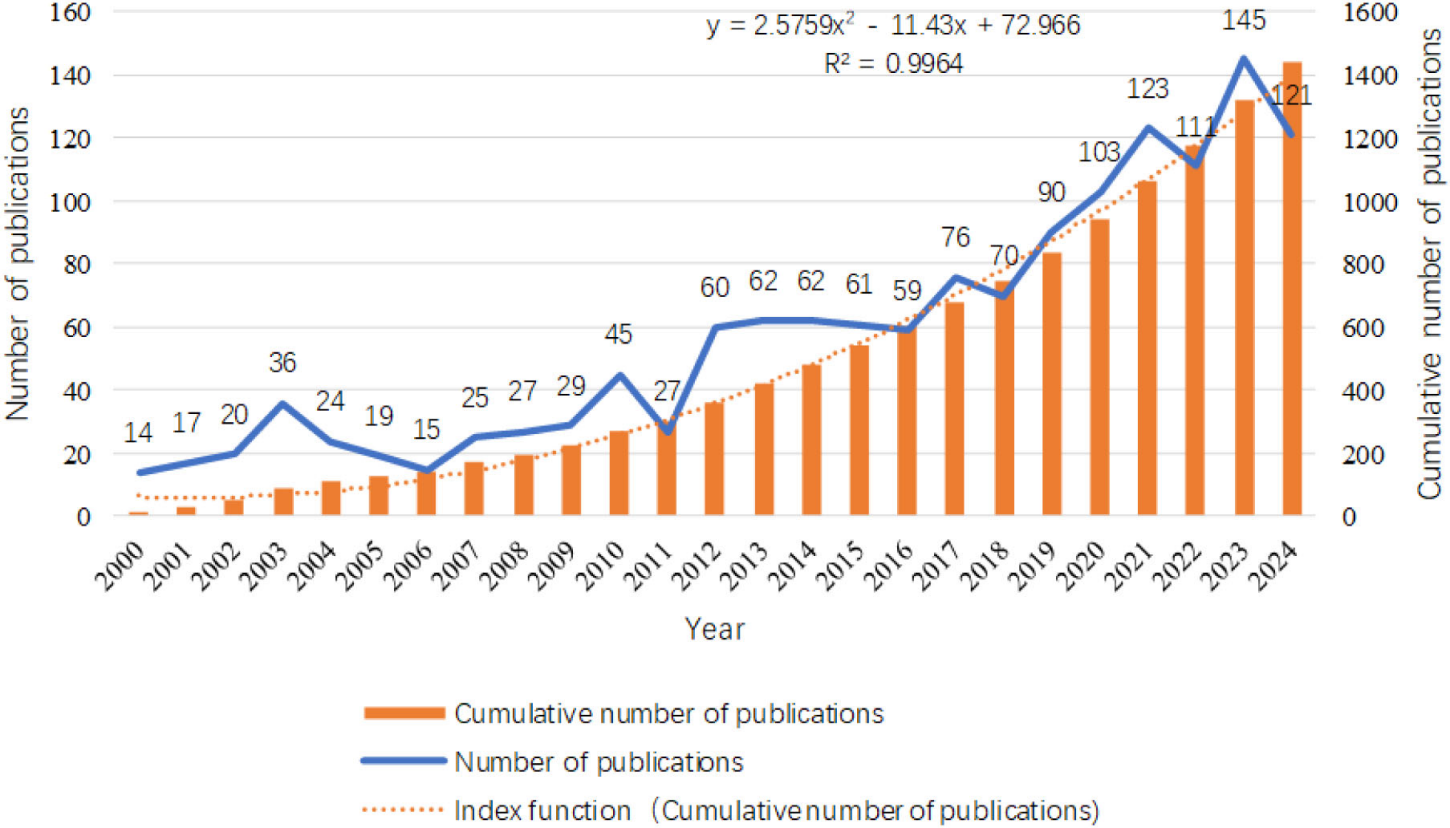
Distribution of the number of publications related to the research on inflammation and thyroid cancer in disease areas.

A comprehensive search was conducted using the Web of Science Core Collection (WOSCC), retrieving a total of 1,441 publications, including 1,169 articles and 272 review articles. As illustrated in **Figure 2**, the chart meticulously depicts the trends in annual publication volume and cumulative publication volume over the past 25 years. Between 2000 and 2008, the average annual publication volume was relatively low, approximately 20 articles, indicating that research on the relationship between inflammation and thyroid carcinoma was still in its foundational stages during this period. However, starting in 2009, the annual publication volume began to exhibit a steady upward trajectory, reaching its peak in 2023 (145 articles). This growth trend underscores the increasing significance of inflammation in thyroid carcinoma research, reflecting a rising academic interest and engagement in this field, with a growing number of researchers dedicating efforts to its exploration. Additionally, we constructed an exponential function model, y = 2.5759x² - 11.43x + 72.966 (R² = 0.9964, where X represents the year and Y represents the cumulative annual publication volume), to describe the trend in cumulative publication volume. The high goodness-of-fit (R² value close to 1) of this model demonstrates its accuracy in capturing and predicting the temporal changes in cumulative publication volume, providing a robust mathematical tool for understanding the research dynamics in this field. Through this model, we can anticipate future publication trends in the study of inflammation and thyroid carcinoma, offering valuable insights for the academic community.

### 3.2 Countries and institutions analysis

**Figure 3:**
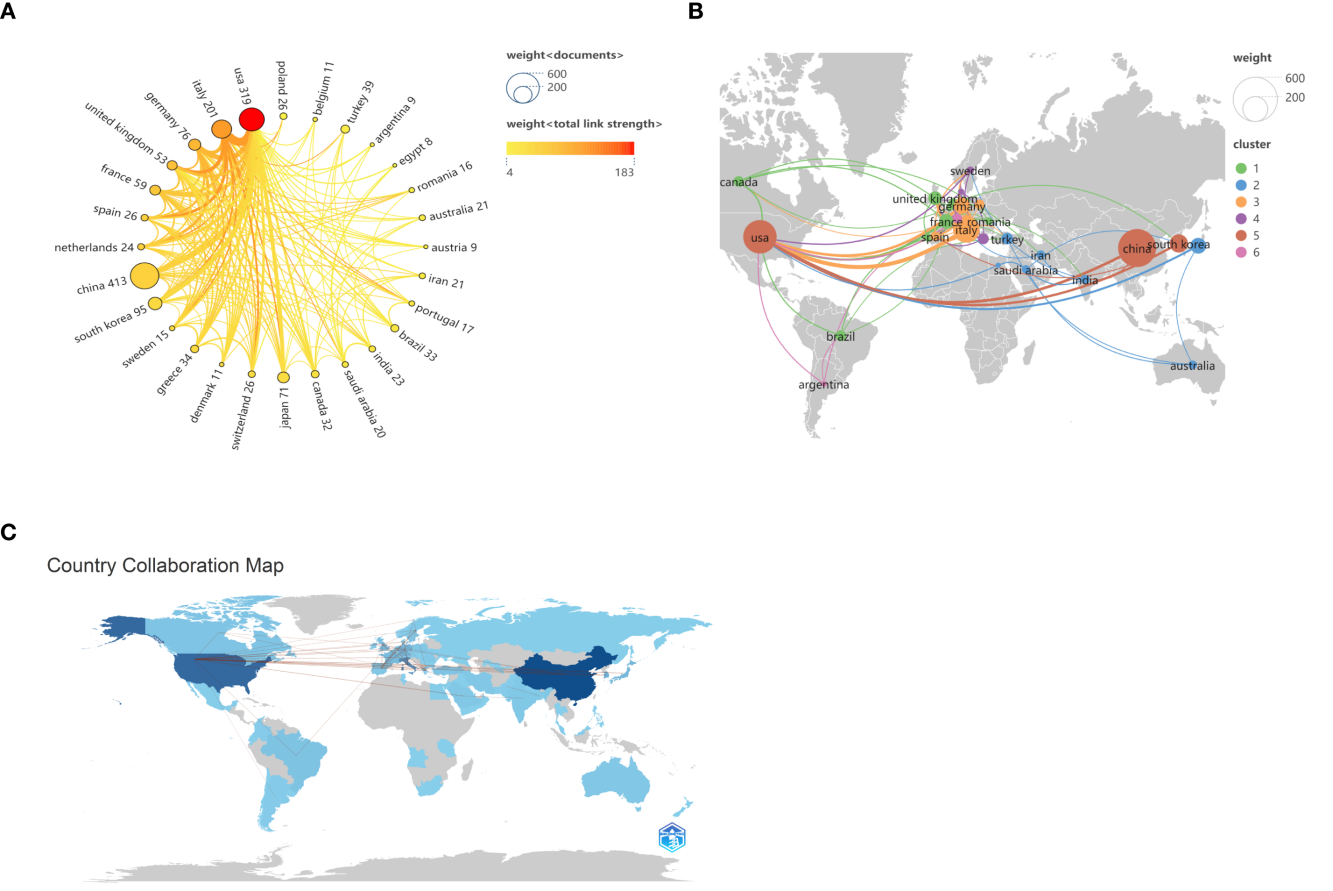
Map of national or regional cooperation. **(A)** Circular chart of national or regional cooperation, the connection between nodes represents cooperation, and the thickness of the line represents the strength of cooperation. **(B)** Map of countries or regions according to the proportion of published papers. **(C)**The contribution of various countries across the world. The darker shades correspond to higher contribution to the field.

From 2000 to 2024, a total of 70 countries globally conducted research related to inflammation and thyroid carcinoma. According to the data provided in **Table 1**, the top three countries by publication volume are China (413 articles, 28.7%), the United States (319 articles, 22.1%), and Italy (201 articles, 13.9%). Other countries with more than 50 articles include South Korea (95 articles, 6.6%), Germany (76 articles, 5.3%), Japan (71 articles, 4.9%), France (59 articles, 4.1%), and the United Kingdom (53 articles, 3.7%). In addition to the number of publications, the citation count of a country’s published articles is also a critical factor in assessing its influence in this research field. Although the United States has fewer publications than China, its citation count (62,809 citations) far exceeds that of China (7,138 citations), making it the most cited country, followed by France (50,536 citations) and Italy (9,224 citations). While France’s publication volume (59 articles) and citation count (50,536 citations) are lower than those of the United States (319 articles, 62,809 citations), its average citation rate (856.54 average citations) is the highest, indicating the qualitative impact of its research contributions. In contrast, China’s high output contrasts sharply with its relatively lower citation count, revealing a need to enhance the quality of research in this potential field. We also analyzed collaborations between different countries and visualized them in **Figures 3A–C**. When the minimum publication threshold was set to 8 articles, 28 countries were included. Nodes represent countries, with the size of each node corresponding to the number of publications by that country in the collaboration network. Lines between nodes indicate collaborative relationships, with the thickness and color intensity of the lines representing the total link strength. Darker and thicker lines denote closer collaborations. The figure shows that China has the largest node, highlighting its significant role in this research field. Although its collaboration strength with other countries is not the highest, it maintains extensive international partnerships. The United States also emerges as a key node, with the highest total collaboration strength (183), particularly with Italy, China, and Germany, among others, with the most intensive collaboration observed with Italy.

**Table 1:** Top 10 countries and with the most documents on research of inflammation in thyroid cancer.

Rank	Country	Documents	Citations	Average Citation
1	China	413	7138	17.28
2	USA	319	62809	196.89
3	Italy	201	9224	45.89
4	South Korea	95	2771	29.17
5	Germany	76	3011	39.62
6	Japan	71	2201	31.00
7	France	59	50536	856.54
8	United Kingdom	53	2218	41.85
9	Turkey	39	541	13.87
10	Greece	34	1475	43.38

**Figure 4:**
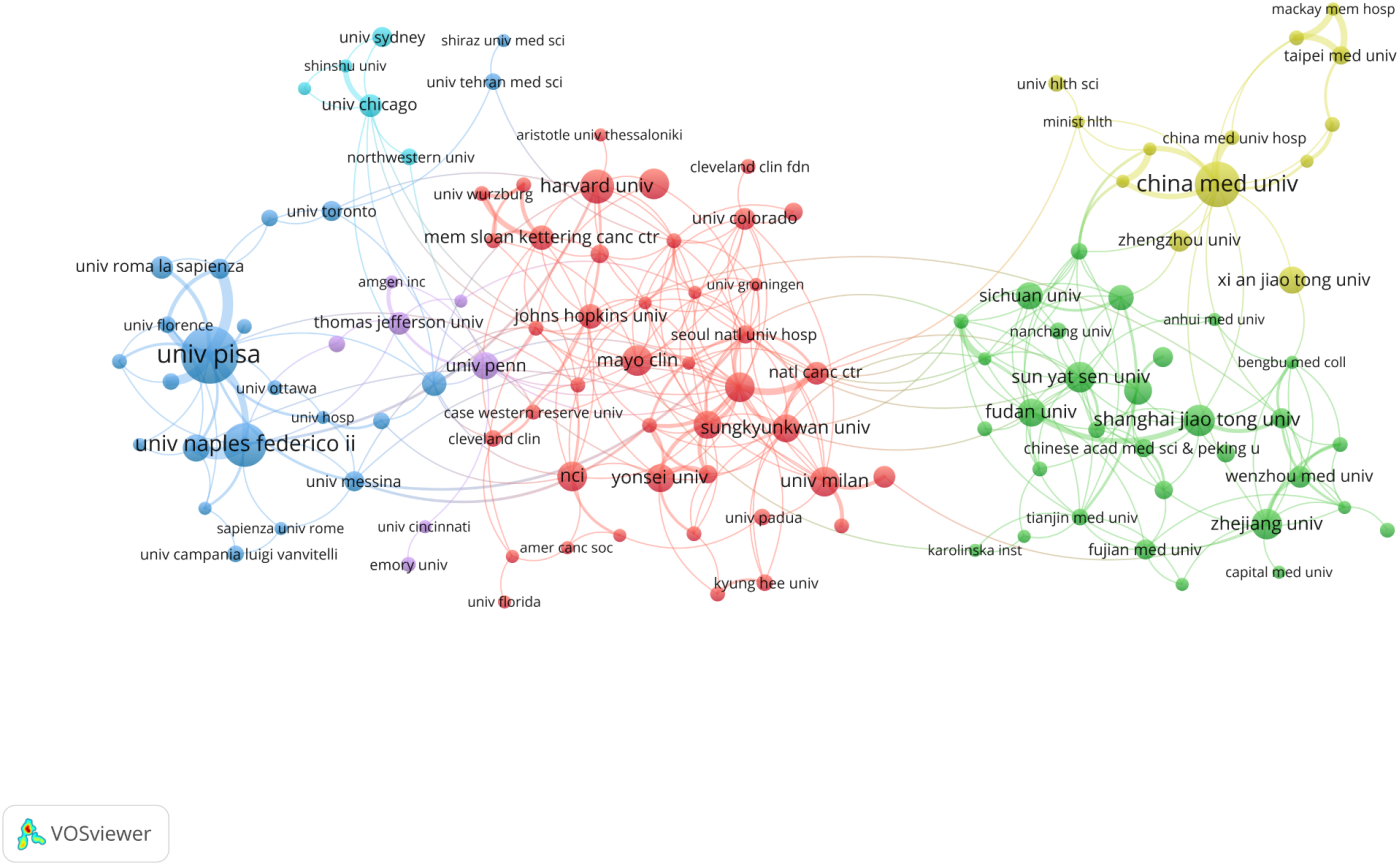
Institutional cooperation map, the size of the nodes indicates the number of publications, and the thickness and length of the links between the nodes indicate the strength and relevance of the connections between the nodes.

A total of 2,054 institutions globally have conducted research on inflammation and thyroid carcinoma. **Table 2** summarizes the top 10 institutions with the highest contributions. The top three institutions with the most publications in this field are the University of Pisa in Italy (N=44), China Medical University in China (N=31), and the University of Naples Federico II in Italy (N=29). Notably, the University of Pisa not only leads in publication volume but also has the highest citation count (3,206 citations), significantly surpassing other institutions. This underscores its leadership in this field. Additionally, among the top 10 institutions, four are from China: China Medical University, Shanghai Jiao Tong University, Sun Yat-sen University, and Zhejiang University. This highlights China’s strong research capabilities, influence, and high level of research activity in this field. When the minimum publication threshold was set to 5 articles, a total of 131 institutions were included in our analysis, forming six distinct clusters, as shown in **Figure 4**. Within these clusters, central nodes such as the University of Pisa, China Medical University, and the University of Naples Federico II play pivotal roles as key hubs, revealing robust collaborative partnerships and academic exchange networks. These central institutions facilitate knowledge exchange and deepen research through frequent collaborations. However, the analysis also reveals that most institutions remain relatively isolated, with limited close collaborations. This fragmented collaboration pattern may hinder the rapid dissemination of knowledge and the efficiency of research. Therefore, strengthening inter-institutional collaborations and building more cohesive academic networks is crucial for advancing research in inflammation and thyroid carcinoma. By fostering cross-institutional partnerships, research progress can be accelerated, and the impact of studies can be enhanced, ultimately leading to greater breakthroughs in scientific development and clinical applications in this field.

**Table 2:** Top 10 institutions with inflammation and thyroid cancer research publications in diseases.

Organization	Documents	Citations	Total link strength	Country
University of Pisa	44	3206	43	Italy
China Medical University	31	1362	25	China
University of Naples Federico II	29	1487	23	Italy
Harvard University	20	1961	11	USA
Shanghai Jiao Tong University	18	356	15	China
Mayo clinic	17	957	17	USA
Sun Yat-sen University	17	297	16	China
University of Athens	17	974	3	Greece
Zhejiang University	17	181	13	China
National Cancer Institute	16	1044	12	USA

### 3.3 Authors and their collaborations

**Figure 5:**
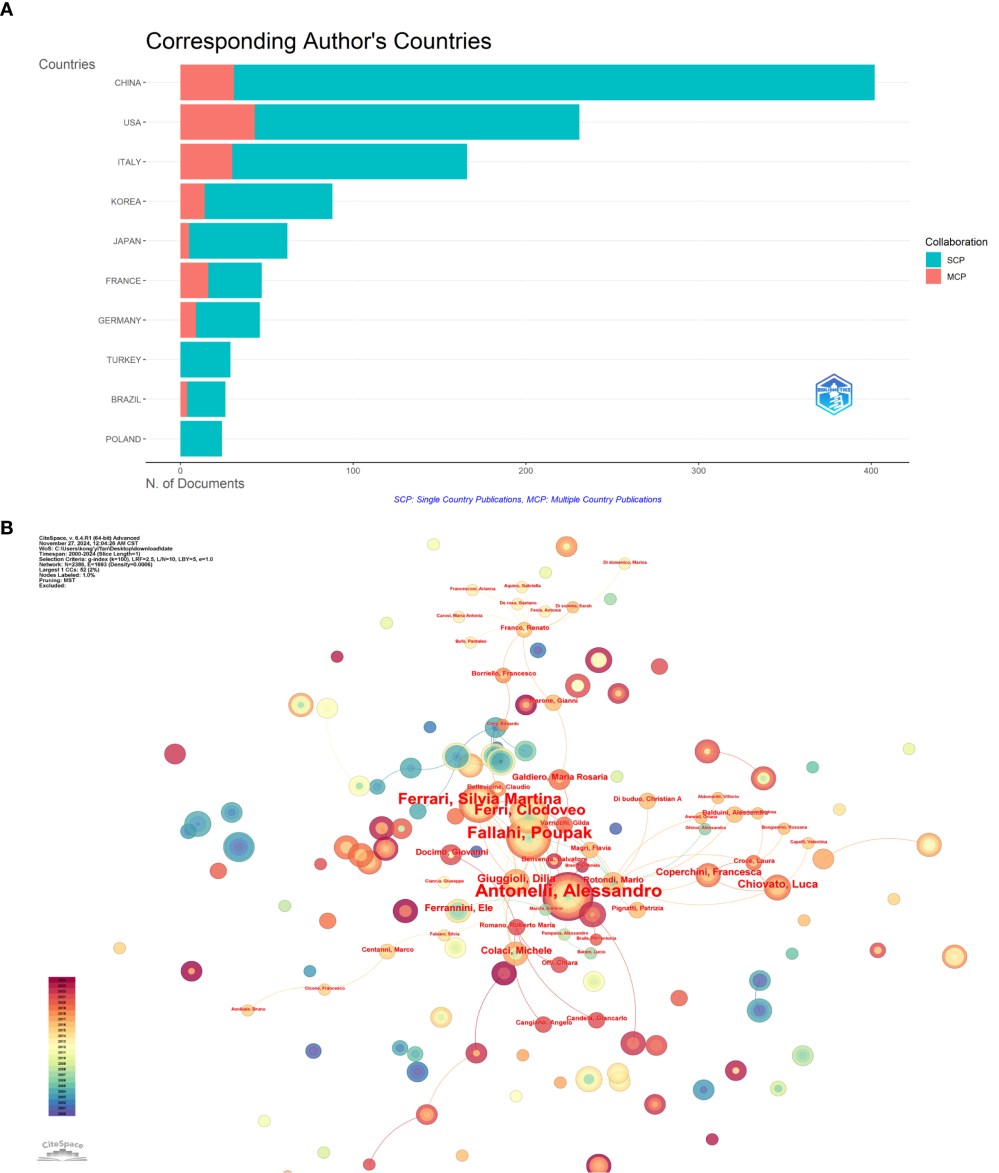
Author-level analysis: **(A)** Corresponding author’s countries, SCP represents the number of co-authored papers by authors of the same nationality, while MCP represents the number of co-authored papers with authors from other countries. **(B)** Co-occurrence of authors network map, nodes represent different authors, node size represents the number of author posts, the thickness of the connecting line between nodes represents the strength of cooperation.

Over the past 24 years, this study has included a total of 8,326 authors. **Table 3** presents the top 10 most prolific authors and their academic contributions in this field. The Italian research team demonstrates remarkable scholarly influence, with Professor Antonelli, Alessandro leading as the most published author (19 articles), accumulating 1,417 citations and achieving an H-index of 15 - all top metrics that underscore his pioneering status. His seminal work *Chemokine (C-X-C motif) ligand (CXCL)10 in autoimmune diseases* has provided fundamental theoretical insights for autoimmune disease research (21). Following closely are Italian scholars Professor Fallahi, Poupak (17 publications) and Professor Ferrari, Silvia Martina (15 publications), whose representative works respectively investigate multisystem involvement in autoimmune thyroiditis (*The association of other autoimmune diseases in patients with autoimmune thyroiditis*) and immune regulation mechanisms within the thyroid cancer microenvironment (*Immune and Inflammatory Cells in Thyroid Cancer Microenvironment*) (22, 23), offering critical perspectives for the field. The scholars ranked fourth to tenth also made outstanding contributions, with their representative works as follows: Professor Ferri, Clodoveo from Italy focused on the relationship between HCV infection and thyroid diseases. His study *Thyroid Involvement in Hepatitis C Virus-Infected Patients with/without Mixed Cryoglobulinemia* provided crucial evidence for the mechanisms of virus-related endocrine disorders (24); Professor Netea-maier, Romana T. from the Netherlands elucidated the regulatory role of autophagy in inflammation in her seminal work, *Modulation of Inflammation by Autophagy: Consequences for Human Disease*, thereby broadening the horizons of metabolism-immune crosstalk research (25).; The study *Inflammatory tumor microenvironment of thyroid cancer promotes cellular dedifferentiation and silencing of iodide-handling genes expression* by Professor Li Zhang’s team in China revealed how inflammatory microenvironments drive thyroid cancer cell dedifferentiation and functional loss, offering potential therapeutic targets for precision treatment (26).; Professor DeGroot, Lj (Japan) pioneered telomerase-targeted gene therapy strategies for anaplastic thyroid carcinoma (*Tumor-specific gene therapy for undifferentiated thyroid carcinoma utilizing the telomerase reverse transcriptase promoter*) (27); Professor Netea, Mihai G. (USA) revealed differential inflammasome activation mechanisms for IL-1β processing in various immune cells (*Differential requirement for the activation of the inflammasome for processing and release of IL-1β in monocytes and macrophages)* (28)*;* Professor Ward, Laura Sterian (Brazil) systematically demonstrated the central role of inflammatory microenvironment in thyroid carcinogenesis (*The role of the inflammatory microenvironment in thyroid carcinogenesis*) *(* 9); and Professor Galdiero, Maria Rosaria (Italy) provided in-depth analysis of tumor-promoting mechanisms by tumor-associated macrophages and neutrophils (*Tumor associated macrophages and neutrophils in tumor progression*) (29). These representative works collectively construct the theoretical framework for thyroid disease and inflammation research, providing crucial references for subsequent studies. **Figure 5A** illustrates the distribution of authors’ nationalities and the extent of international collaboration. SCP (Single Country Publications) represents the number of co-authored papers by authors from the same country, while MCP (Multiple Country Publications) denotes the number of co-authored papers with authors from other countries. The SCP of the top 10 countries by publication volume is higher than their MCP, particularly in China, the United States, and Italy, where the contrast is most pronounced. This suggests that their research collaborations are predominantly domestic, with limited international cooperation. **Figure 5B** presents a visualization of the author collaboration network, revealing a relatively dispersed distribution of authors with only limited connections identifiable in the network, indicating a moderate overall collaboration intensity. Among these authors, Antonelli, Alessandro stands out as a central figure due to his substantial publication volume and high citation count. His average of 74.6 citations per article not only reflects the impact of his research but also underscores his academic contributions to the field.

**Table 3:** The top 10 productive authors in the field of inflammation in thyroid cancer.

Author	Publications	Citations	Total link strength	Average citations per publication	H-index
Antonelli, Alessandro	19	1417	73	74.6	15
Fallahi, Poupak	17	1346	68	79.2	13
Ferrari, Silvia Martina	15	1249	61	83.3	12
Ferri, Clodoveo	12	991	45	82.6	9
Netea-maier, Romana T.	9	289	34	32.1	8
Zhang, Li	9	224	19	24.9	6
Degroot, Lj	8	168	8	21	8
Netea, Mihai G.	8	281	32	35.1	7
Ward, Laura Sterian	8	281	8	35.1	6
Galdiero, Maria Rosaria	7	424	29	60.6	6

Antonelli, Alessandro’s research primarily focuses on several key areas (1): Investigating the link between autoimmune diseases and thyroid carcinoma: Thyroid autoimmunity and thyroid cancer, particularly papillary thyroid carcinoma (PTC), can coexist, although the exact mechanisms remain unclear. The increased incidence of thyroid cancer coincides with the rise in autoimmune thyroid diseases (AITD), suggesting an association between these pathologies. Additionally, elevated TSH levels and thyroid autoimmunity are considered independent risk factors for thyroid cancer. Future research is necessary to elucidate the connection between thyroid autoimmunity and cancer, which will aid in designing personalized therapies for TC patients with AITD (2). Immune cells and inflammatory cells in the thyroid cancer microenvironment: The ability of tumor cells to evade immune system destruction is a hallmark of cancer. Immune cells within the tumor microenvironment (TME) promote tumor growth by secreting cytokines and chemokines, while cancer cells suppress immune responses through various mechanisms, evading immune surveillance. Understanding the molecular and immunological characteristics of the TME is crucial for developing diagnostic and therapeutic strategies for thyroid carcinoma, particularly immunotherapies such as immune checkpoint inhibitors, which can activate the immune system to target cancer cells. These studies provide valuable insights into the complex pathology of thyroid cancer and offer a scientific foundation for developing novel therapeutic strategies. Through these high-quality research efforts, Antonelli, Alessandro has played a pivotal role in advancing the field of inflammation and thyroid carcinoma research.

### 3.4 Journals and co-cited journals analysis

**Figure 6:**
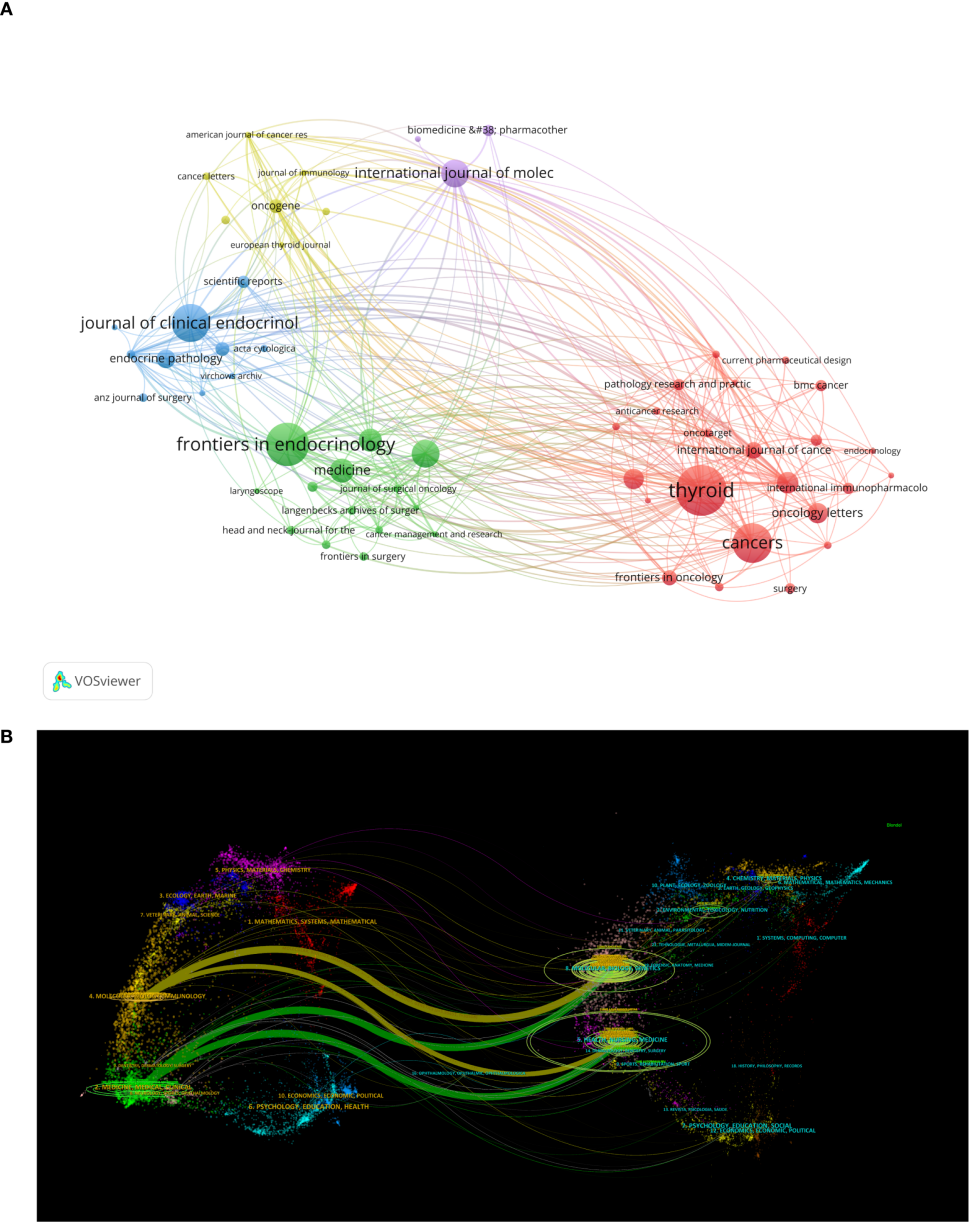
**(A)** Showcases visualizations of journals;**(B)** The dual-map overlay of journals, the left label in the figure represents citing journals, the right label represents cited journals, and the colored paths represent the citation relationships between them.

Journals serve as vital mediums for disseminating and exchanging academic knowledge. All papers related to this study were published in 625 journals. **Table 4** lists the top 10 journals with the highest publication volume in this field. *Thyroid* ranks first with 40 articles, an impact factor of 1.56, and a Journal Citation Reports (JCR) division of Q1 (journals within the top 25% of impact factors). This peer-reviewed journal publishes original articles and reviews reflecting the ever-expanding activities in the thyroid field, ranging from the molecular biology of the cell to the clinical management of thyroid disorders. As the official journal of the American Thyroid Association, it is recognized as the most authoritative journal in the field of thyroid diseases. Ranking second is *Frontiers in Endocrinology* with 34 publications and an impact factor of 0.87. This journal primarily advances our understanding of the endocrine system and publishes research on novel therapies for prevalent health issues such as obesity, diabetes, reproductive system disorders, and aging endocrinology. **Figure 6A**, created using the VOSviewer tool, visualizes the interconnections and publication volumes of journals that have published at least five related articles. This visualization provides an intuitive platform for observing and analyzing collaborative and competitive relationships among journals. **Figure 6B** further deepens this analysis by presenting a dual-map overlay, illustrating the interaction between citing journals and cited journals. The left side of the map represents citing journals, typically reflecting the latest research trends and knowledge frontiers in a field, while the right side represents cited journals, which form the foundational knowledge base of the field. The map clearly shows that citing literature is concentrated in two domains: (1) Medicine/Medical/Clinical and (2) Molecular/Biology/Immunology. Correspondingly, cited journals are also primarily focused on two domains: (1) Health/Nursing/Medicine and (2) Molecular/Biology/Genetics. This distribution reveals the knowledge structure and research focus within the field of inflammation and thyroid carcinoma. Additionally, the main journal pathways in the map exhibit a cross-pattern, forming four primary citation paths, represented by two distinct colors. The yellow path indicates that journals in the Molecular/Biology/Immunology domain cite literature from the Molecular/Biology/Genetics and Health/Nursing/Medicine domains. The green path shows that journals in the Medicine/Medical/Clinical domain also cite literature from the Molecular/Biology/Genetics and Health/Nursing/Medicine domains. These pathways not only demonstrate citation relationships among journals but also reflect the mutual influence and knowledge flow between different research domains. Through this in-depth visualization analysis, we can better understand the academic network within the field of inflammation and thyroid carcinoma research, as well as the roles and contributions of individual journals.

**Table 4:** The top 10 productive journals related to research of inflammation in thyroid cancer.

Journal	Publications	IF (JCR2023)	JCR quartile
Thyroid	40	1.56	Q1
Frontiers In Endocrinology	34	0.87	Q2
Cancers	31	0.91	Q1
Journal of Clinical Endocrinology & Metabolism	30	1.25	Q1
endocrine	22	0.72	Q2
international journal of molecular sciences	22	0.71	Q1
Medicine	19	0.35	Q2
Plos One	18	0.88	Q1
Endocrine-Related Cancer	17	0.88	Q2
Journal of Endocrinological Investigation	16	0.95	Q2

**Table 5** lists the top 10 journals by co-citation frequency. The *Journal of Clinical Endocrinology & Metabolism* ranks first with the highest number of citations (N=1,940), an impact factor of 1.25, and a Journal Citation Reports (JCR) division of Q1. Additionally, among the top 10 journals by co-citation frequency, the *New England Journal of Medicine* (NEJM) has the highest impact factor (IF=25.31). The top three journals by co-citation frequency have publication counts of 1,940, 1,871, and 1,382, significantly surpassing other journals.

**Table 5:** The top 10 co-cited journals related to research of inflammation in thyroid cancer.

Cited Journal	Citations	IF (JCR2023)	JCR quartile
Journal of Clinical Endocrinology & Metabolism	1940	1.25	Q1
Thyroid	1871	1.56	Q1
Cancer Research	1382	1.99	Q1
Oncogene	800	1.47	Q1
Plos One	793	0.88	Q1
Proceedings of the National Academy of Sciences of the United States of America	779	2.4	Q1
Nature	746	11.1	Q1
The New England Journal of Medicine	720	25.31	Q1
Journal of Biological Chemistry	698	0.85	Q2
Clinical Cancer Research	647	2.52	Q1

### 3.5 Co-cited references and reference with citation bursts

**Figure 7:**
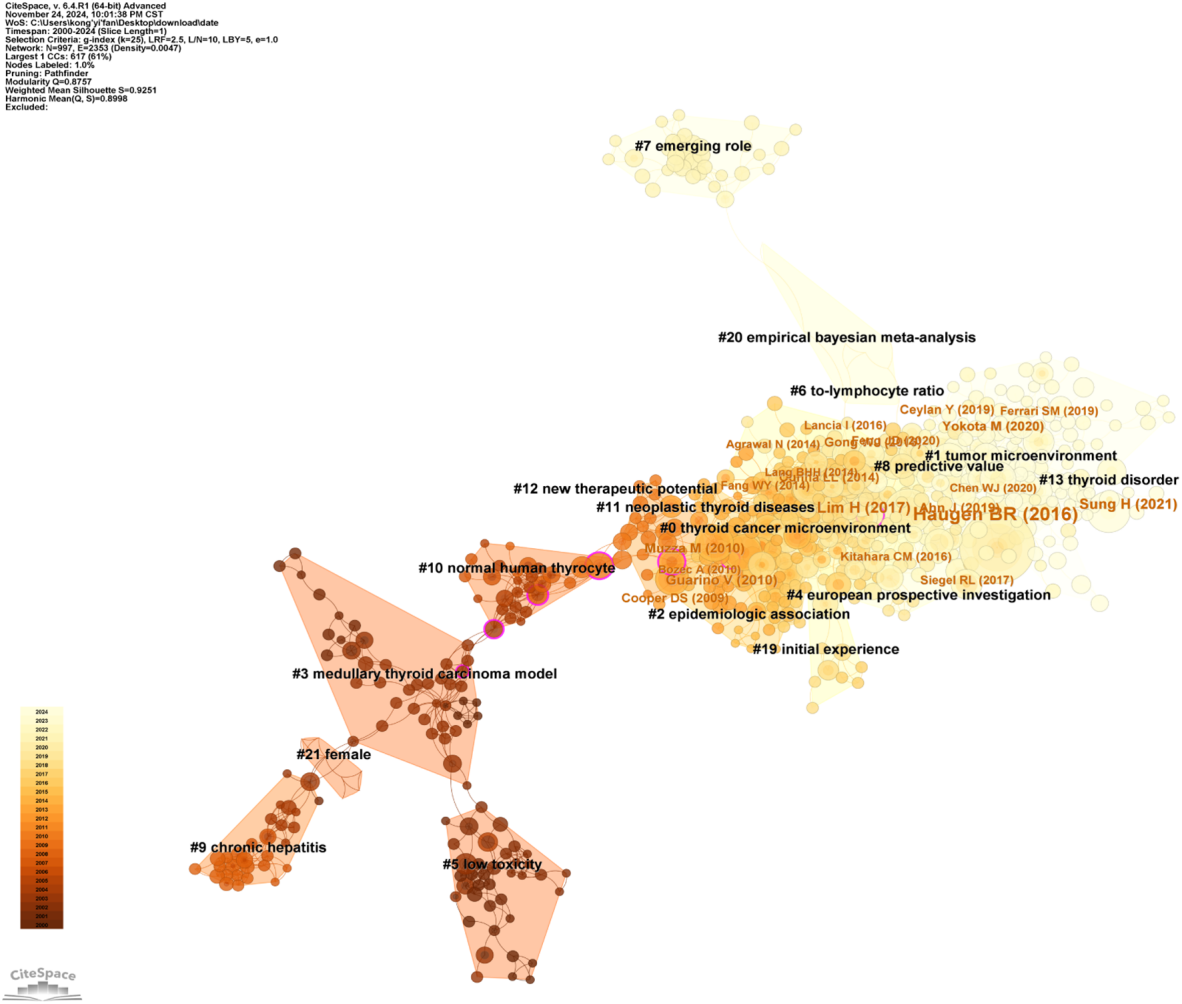
A timeline view for co-cited references.

The study in this field included 1,441 articles, encompassing a total of 59,808 cited references. **Table 6** lists the top 10 most cited references, with publications by Bryan R. Haugen (30), Lisa M. Coussens (31), and Valentina Guarino (32) as first authors ranking in the top three, with citation counts of 138, 75, and 72, respectively. **Figure 7** provides an intuitive visualization of the co-citation network analysis of references, revealing the structural characteristics and clustering effects of the network. The analysis results indicate that the Modularity Q of the network reached 0.8757, a value close to 1, demonstrating a highly significant modular structure within the network. Additionally, the average Silhouette S was as high as 0.9251, reflecting the superior clustering effectiveness of the network, meaning that the references were effectively grouped into highly similar clusters. These metrics collectively confirm the efficiency and accuracy of the co-citation network analysis, providing a powerful tool for understanding the relationships among references and the knowledge structure of the research field. Furthermore, the network can be divided into 17 clusters, with the largest being Cluster #0: thyroid cancer microenvironment (33–37). The earliest cluster to initiate research in this field was Cluster #5: low toxicity (38–42), while Cluster #7: merging role (43–45), Cluster #8: predictive value (46–50), and Cluster #13: thyroid disorder (2, 51–53) represent recent research hotspots, indicating a growing interest and exploration of this field by researchers.

**Table 6:** Ranking of the top 10 co-cited references for inflammation in thyroid cancer.

Reference	Citations	Journals	First author	Year
2015 American Thyroid Association Management Guidelines for Adult Patients with Thyroid Nodules and Differentiated Thyroid Cancer: The American Thyroid Association Guidelines Task Force on Thyroid Nodules and Differentiated Thyroid Cancer (30)	138	THYROID	Bryan R Haugen	2016
Inflammation and cancer (31)	75	NATURE	Lisa M Coussens	2002
Thyroid cancer and inflammation (32)	72	MOL CELL ENDOCRINOL	Valentina Guarino	2010
The tight relationship between papillary thyroid cancer, autoimmunity and inflammation: clinical and molecular studies (109)	58	CLIN ENDOCRINOL	Marina Muzza	2010
Inflammation and cancer: back to Virchow? (7)	56	LANCET	F Balkwill	2001
Revised American Thyroid Association management guidelines for patients with thyroid nodules and differentiated thyroid cancer (110)	56	THYROID	American Thyroid Association (ATA) Guidelines Taskforce on Thyroid Nodules and Differentiated Thyroid Cancer	2009
Increased density of tumor-associated macrophages is associated with decreased survival in advanced thyroid cancer (111)	56	ENDOCR-RELAT CANCER	Mabel Ryder	2008
Cancer-related inflammation (112)	52	NATURE	Alberto Mantovani	2008
Increasing incidence of thyroid cancer in the United States, 1973-2002 (113)	51	JAMA-J AM MED ASSOC	Louise Davies	2006
Hallmarks of cancer: the next generation (114)	51	CELL	Douglas Hanahan	2011

**Figure 8:**
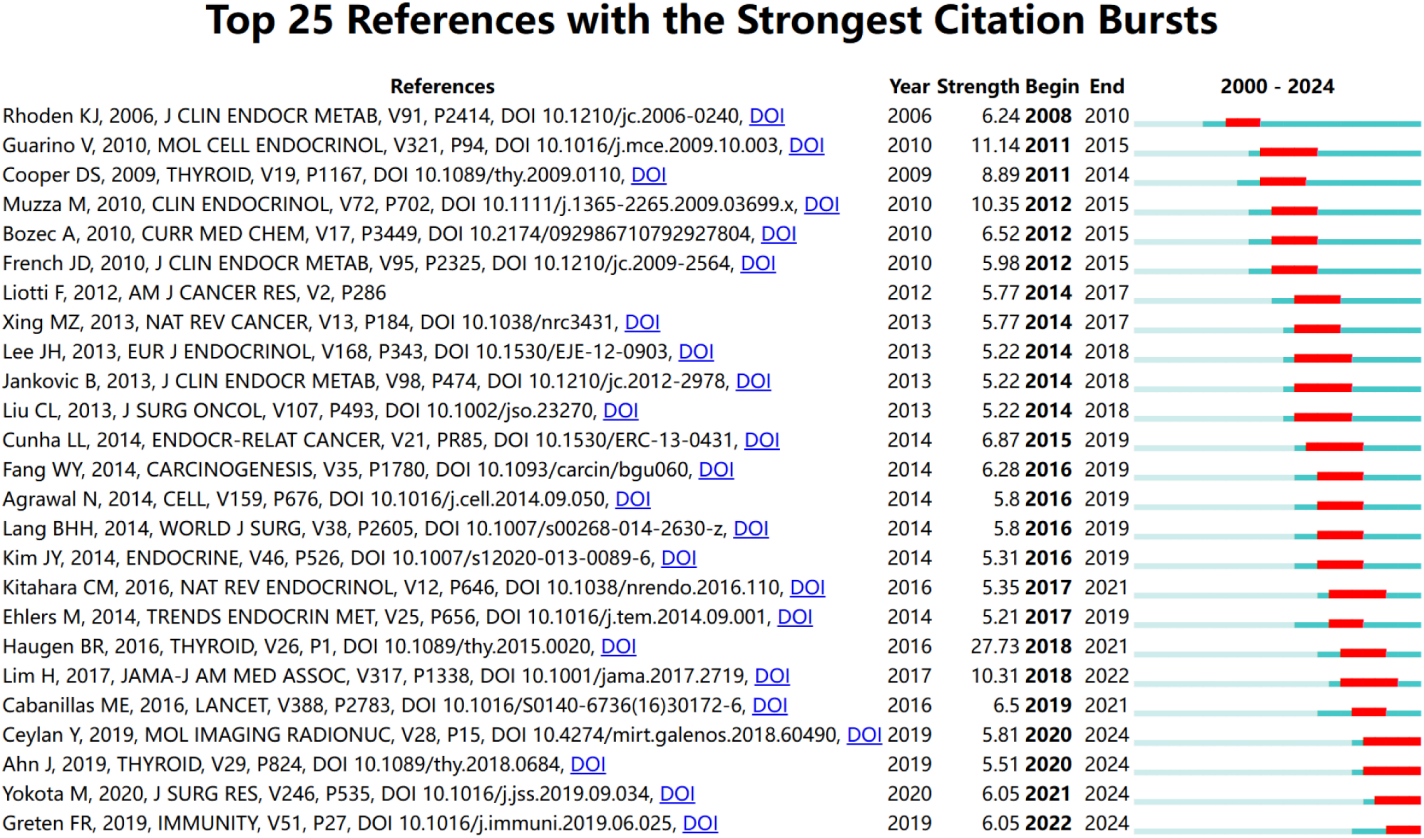
The most often cited top 20 sources. Citations that were particularly high that year are shown in red.

**Figure 8** provides an in-depth analysis of the top 25 references with the strongest citation bursts, enabling the precise identification of publications that experienced a sharp increase in citations during specific time periods, thereby revealing research hotspots within those intervals. The phenomenon of citation bursts in this field began in 2006 and continues to the present, with many references still being widely cited. This indicates that research on the association between thyroid carcinoma and inflammation remains a focal point in recent years and is likely to persist in the coming years. Among these references, the study with the highest citation burst strength is the work by Bryan R. Haugen as the first author, titled *2015 American Thyroid Association Management Guidelines for Adult Patients with Thyroid Nodules and Differentiated Thyroid Cancer: The American Thyroid Association Guidelines Task Force on Thyroid Nodules and Differentiated Thyroid Cancer* (30). Its citation burst strength reached 27.73, with a significant surge in citations from 2016 to 2021. The duration of these citation bursts spans 5 to 6 years, with burst strengths ranging from 5.22 to 27.73. This not only highlights the vibrancy of research in this field but also underscores the continuous evolution and development of research dynamics. These data provide profound insights into the key scientific contributions and knowledge advancements within the field of thyroid carcinoma and inflammation research.

### 3.6 The co-occurrence and clustering of keywords

**Figure 9:**
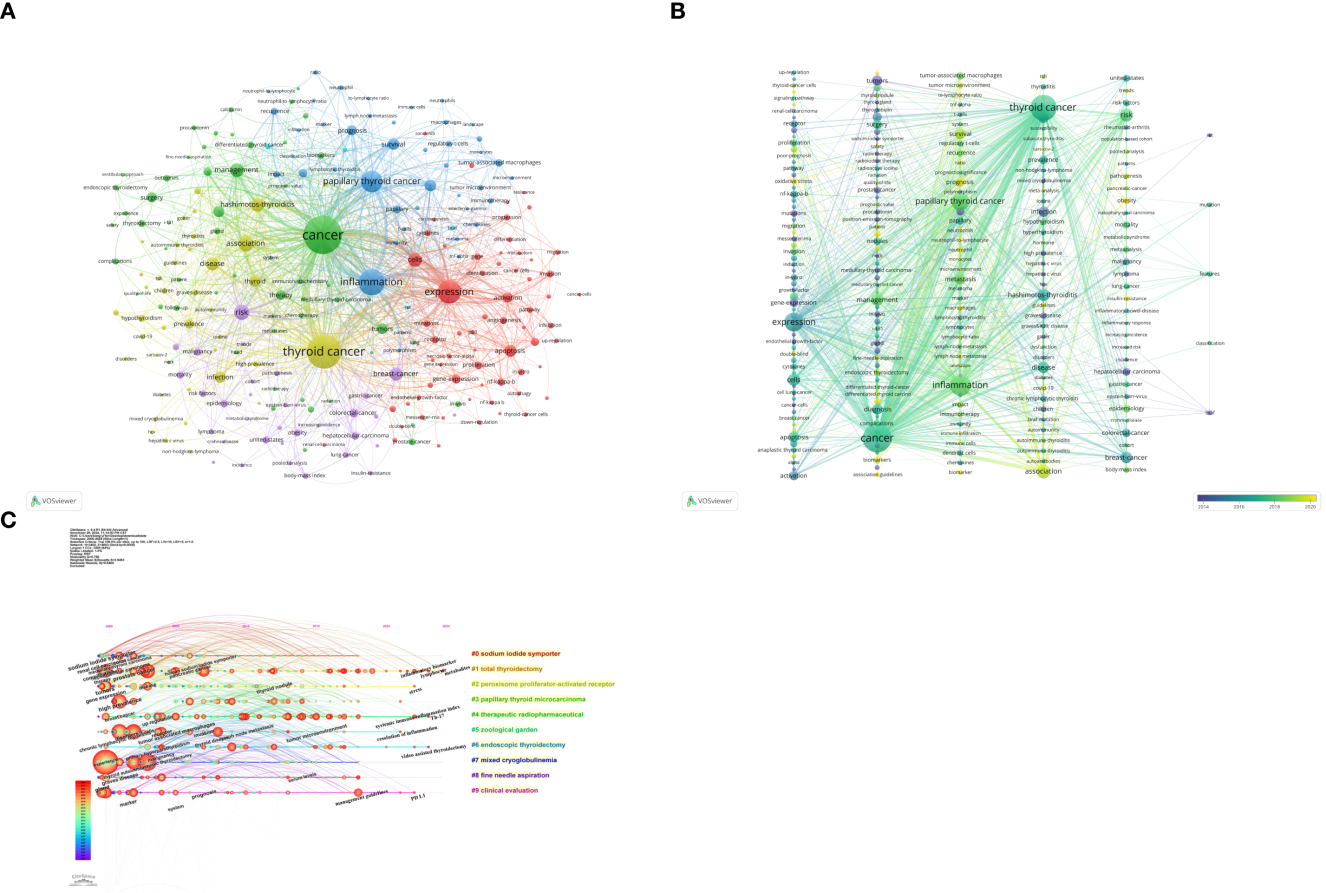
Cluster analysis and topic evolution of hot topics: **(A)** Network map of keywords occurring more than 10 times. **(B)** Overlay map of keywords based on average publication year. **(C)** Timeline view of keyword clustering, in the timeline view, the keywords on the same horizontal line belong to the right cluster. The colors of lines and keywords in the view correspond to the colors of the time bar in the lower left corner.

The 1,441 articles in this study encompassed a total of 6,340 keywords. Using VOSviewer, after merging synonyms and setting a minimum occurrence threshold of 10 times per keyword, 239 keywords were included in the visualization analysis (**Figures 9A, B**), integrating keyword contributions. In the visualization map, each color represents a cluster, and each node represents a keyword. The size of the node indicates the frequency of the keyword’s occurrence, with larger nodes representing more prominent research hotspots in the field. Lines between nodes denote co-occurrence relationships between two keywords. The top 10 keywords by frequency include *cancer* (520 occurrences), *thyroid cancer* (431 occurrences), *inflammation* (272 occurrences), *expression* (232 occurrences), *papillary thyroid cancer* (202 occurrences), *association* (116 occurrences), *risk* (112 occurrences), *cells* (97 occurrences), *management* (96 occurrences), and *Hashimotos-thyroiditis* (94 occurrences). These keywords represent the primary research themes in thyroid carcinoma and inflammation from 2000 to 2024. The keywords were divided into six clusters, with the top five clusters being: the first cluster (red), focusing on the expression of inflammation-related factors in thyroid carcinoma to explore the role of inflammation in its development; the second cluster (green), investigating the link between inflammation and the management and diagnosis of thyroid carcinoma; the third cluster (blue), related to the tumor microenvironment of thyroid carcinoma; the fourth cluster (yellow), studying the association between Hashimoto’s thyroiditis and thyroid carcinoma; and the fifth cluster (purple), exploring the link between obesity and thyroid carcinoma risk. Using CiteSpace for clustering analysis, 10 clusters were generated, and a timeline view reflecting the progression of keywords over time was created (**Figure 9C**). Each color represents a different cluster, and each cluster signifies a key theme in the field: Cluster #0 *sodium iodide symporter*, Cluster #1 *total thyroidectomy*, Cluster #2 *peroxisome proliferator-activated receptor*, Cluster #3 *papillary thyroid microcarcinoma*, Cluster #4 *therapeutic radiopharmaceutical*, Cluster #5 *zoological garden*, Cluster #6 *endoscopic thyroidectomy*, Cluster #7 *mixed cryoglobulinemia*, Cluster #8 *fine needle aspiration*, and Cluster #9 *clinical evaluation*. The Modularity Q of this module was 0.756, and the mean Silhouette S was 0.9463. These 10 clusters span topics from *sodium iodide symporter* to *clinical evaluation*. From a chronological perspective, early research on thyroid carcinoma and inflammation focused on keywords such as *thyroid autoimmunity*, *complications*, *gene expression*, *high prevalence*, and *chronic lymphocytic thyroiditis*. Recent trends, however, revolve around *metabolites*, *lymphocyte*, *inflammatory biomarker*, *stress*, *Th-17*, *systemic immune-inflammation index*, *resolution of inflammation*, *video-assisted thyroidectomy*, *management guidelines*, and *PD-L1*. Among these, *Th-17* and *systemic immune-inflammation index* are two of the most frequently studied keywords in recent years. This highlights potential future research directions and hotspots in the field, while also contributing to the advancement and innovative development of scientific research in this area.

## 4 Discussion

With the advent of the big data era, researchers must thoroughly grasp the latest advancements in their fields. Bibliometric analysis, distinct from systematic reviews and meta-analyses, utilizes visualization tools such as VOSviewer and CiteSpace to conduct comprehensive analyses of existing literature, intuitively revealing research trends and predicting potential future hotspots. This approach provides researchers with a powerful tool to discern dynamic changes and emerging directions in academic domains (54). This study employs bibliometric analysis to summarize the role of inflammation in the development and progression of thyroid carcinoma, offering an intuitive perspective on the field’s trends and future research priorities.

### 4.1 General information related to publications

Relying on the Web of Science database, a comprehensive literature search was conducted to gather research on inflammation and thyroid carcinoma from 2000 to 2024. After a rigorous screening process, 1,441 articles were selected, originating from 70 countries, authored by 8,326 researchers from 2,054 institutions, and published in 625 academic journals. These articles encompass 59,808 references and 6,340 keywords, providing a comprehensive research perspective and rich data resources.

From the research results, the number of publications in this field has shown a steady annual increase, indicating that inflammation and thyroid carcinoma have become focal points of attention. Although China is the only developing country among the top 10 most productive nations, analysis of the national network distribution reveals that China ranks first in publication volume, accounting for 28.7% of global publications. This underscores China’s central role in inflammation and thyroid carcinoma research. This prominence may be attributed to the rising incidence and mortality of thyroid carcinoma in China, driven by lifestyle changes and an aging population, which has heightened the demand and urgency for related research.

Among the top 10 institutions by publication volume, the University of Pisa in Italy leads with outstanding research achievements and academic contributions, reflecting its leadership in the field and its pivotal role in global research collaboration. Notably, four of these top institutions are located in China, highlighting China’s significant advantage in publication volume and research influence, as well as its rapid rise and growing competitiveness on the global academic stage. This is attributed to China’s strengths: 1) A vast pool of researchers and medical professionals, providing abundant human resources for medical research. 2) Continuous increases in funding from the Chinese government and various institutions, offering robust financial support for scientific endeavors. 3) Significant investments in research facilities and experimental equipment, providing advanced infrastructure for medical research. 4) Encouragement of interdisciplinary collaboration, fostering innovation at the intersection of medicine and fields such as bioinformatics and materials science.5) Remarkable progress in translating medical research into clinical applications and industrial advancements, driving the evolution of medical technologies and the healthcare industry.

In our in-depth analysis of the author community, we observed that Antonelli, Alessandro stands out with a notable publication volume (N=19), establishing himself as one of the most active researchers in the field. This achievement not only reflects his extensive academic experience but also underscores the widespread recognition and citation of his research within the academic community. Such influence and recognition serve as strong evidence of his academic contributions and professional expertise. Further examination of the collaboration network among authors reveals an intriguing pattern: collaborations are predominantly regional, with most partnerships confined within national borders, and domestic collaborations being particularly robust. In contrast, international collaborations are relatively scarce. This pattern may be influenced by cultural, linguistic, resource allocation, and research policy factors. However, this limitation also highlights significant potential for expanding cross-border collaborations. To advance research on thyroid carcinoma and inflammation, we recommend that countries and authors with established close collaborations continue to strengthen their academic exchanges. Simultaneously, efforts should be made to build academic connections with countries and authors with fewer collaborations, fostering high-quality multicenter studies and promoting the globalization of thyroid carcinoma and inflammation research.

### 4.2 Research foundations

Impact factor (IF), Journal Citation Reports (JCR) category quartiles, and total citation counts are three key metrics for assessing a journal’s academic influence. In the field of endocrinology, particularly in publications related to thyroid carcinoma and inflammation, the following journals have demonstrated exceptional performance, ranking among the top ten: *Thyroid* leads with 40 publications, an impact factor of 1.56, and a JCR Q1 ranking, showcasing its leading position in the field. *Frontiers in Endocrinology* follows closely, having published 34 articles, with an impact factor of 0.87 and a JCR Q2 ranking, indicating its high academic value in endocrinology. *Cancers* has published 31 articles, with an impact factor of 0.91 and a JCR Q1 ranking, highlighting its influence in oncology. Particularly noteworthy is the *Journal of Clinical Endocrinology & Metabolism*, which, although not the highest in publication volume, boasts a total citation count of 1,940, an impact factor of 1.25, and a JCR Q1 ranking. This not only reflects the journal’s authority in endocrinology and metabolism but also underscores its significant global influence. Looking ahead, these journals will undoubtedly remain premier platforms for publishing high-quality research in thyroid carcinoma and inflammation. As research continues to advance, it is anticipated that more academic articles on inflammation and thyroid-related topics will be prioritized in these journals, further driving academic progress and international exchange in the field.

Co-cited references refer to publications that are cited together across multiple studies, forming the foundational core of knowledge within a specific research domain. This co-citation relationship elucidates the interconnectedness and academic influence among research works. In the field of thyroid carcinoma and thyroid nodule research, the article by Bryan R. Haugen et al., published in 2016 in *Thyroid*—*2015 American Thyroid Association Management Guidelines for Adult Patients with Thyroid Nodules and Differentiated Thyroid Cancer: The American Thyroid Association Guidelines Task Force on Thyroid Nodules and Differentiated Thyroid Cancer (* 30)—has emerged as the most cited reference in the field over the past 24 years. This distinction not only highlights the authoritative standing of this publication in thyroid disease management guidelines but also underscores its profound impact on clinical practice and academic research. The extensive citation of this work attests to its pivotal role in the therapeutic guidelines for thyroid nodules and differentiated thyroid carcinoma, as well as its critical contribution to advancing knowledge and fostering international collaboration within this field.

### 4.3 Identifying emerging themes and research hotspots

Keyword co-occurrence analysis using VOSviewer is an effective methodology for uncovering the primary research directions and hotspots within a specific academic field. In this study, we extracted 6,340 keywords from the literature, of which 239 were identified as high-frequency keywords due to their occurrence in more than 10 publications. These high-frequency keywords not only represent the commonly used terminology in the research domain but also reflect its focal points. Through clustering analysis, these 239 high-frequency keywords were further categorized into six distinct clusters, each representing a specific theme or research direction within the field. This clustering approach not only aids in identifying and understanding key concepts within the research domain but also reveals potential connections and interactions among different research directions. By employing this method, researchers can more clearly grasp the dynamics of the field, uncover new research opportunities, and provide guidance for future research directions. The primary themes of the top five clusters are as follows:

The first cluster primarily investigates the expression of inflammation-related factors in thyroid carcinoma to explore the role of inflammation in its developmental mechanisms, thereby summarizing therapeutic strategies and prognostic predictions. Key inflammation-related factors include the IL-1 family, IL-6, IL-8, TNF-α, chemokines, NF-κB, UHRF1, and EGR1. These factors play significant roles in the pathogenesis and metastasis of thyroid carcinoma. IL-1α and IL-1β, encoded by the IL1A and IL1B genes, promote inflammatory responses and tumor progression in thyroid carcinoma. Studies have shown that IL1A gene polymorphisms (e.g., rs3783521, rs3783546) are significantly associated with thyroid carcinoma risk, particularly in male patients (55). Interleukin-6 (IL-6) and IL-8 promote tumor angiogenesis and metastasis by activating the STAT3 and NF-κB pathways. Elevated IL-8 levels are correlated with lymph node metastasis and may serve as a prognostic marker (56). Tumor necrosis factor-α (TNF-α) is highly expressed in the thyroid carcinoma microenvironment and enhances cancer cell invasiveness by regulating pathways such as matrix metalloproteinases (MMPs). Clinical data indicate that serum TNF-α levels are significantly elevated in thyroid carcinoma patients and are associated with tumor staging (57). TNF-α also exhibits cytostatic effects and acts as a growth inhibitor for thyroid carcinoma cells (58). Additionally, the chemokine network plays a crucial role in thyroid carcinoma progression. Thyroid carcinoma cells secrete chemokines such as CCL2 and CXCL8 to recruit immunosuppressive cells (e.g., TAMs), creating an immune escape microenvironment (59). In thyroid carcinoma cell lines, different markers are expressed depending on the degree of tumor differentiation. NF-κB, a major player in inflammation, regulates the expression and function of various chemokines and cytokines in inflammatory cells. By stimulating the production of pro-inflammatory cytokines in tumor-infiltrating immune cells, NF-κB promotes thyroid carcinoma initiation and metastasis. Therefore, drugs targeting NF-κB activity may improve chemotherapy and radiotherapy outcomes for thyroid carcinoma (60). Furthermore, the expression of inflammation-related factors in thyroid carcinoma can also facilitate metastasis. For example, inflammatory cytokines downregulate EGR1 expression, leading to a significant reduction in LNCRTCTS, a tumor-suppressive lncRNA controlled by EGR1. LNCRTCTS significantly inhibits the proliferation and metastasis of PTC cells both *in vivo* and *in vitro*. Understanding the mechanisms of thyroid carcinoma metastasis is crucial for developing novel clinical therapies to improve outcomes in advanced PTC (61). Moreover, UHRF1, an oncogene that promotes cancer cell development, induces c-Jun/AP-1 activation and transcription of inflammation/metastasis-related cytokines, facilitating cancer cell migration and invasion. Targeting this pathway may offer new therapeutic strategies for thyroid carcinoma patients (62). Inflammation-associated mediators orchestrate tumor progression through dual mechanisms: (1) by activating pivotal signaling pathways that enhance neoplastic proliferation, suppress apoptosis, and bolster cellular survival—effectively promoting the expansion of tumor-sensitive cells; and (2) by facilitating angiogenesis, epithelial-mesenchymal transition (EMT), and immune evasion, thereby accelerating malignancy and enhancing drug resistance. The chronic inflammatory microenvironment substantially elevates recurrence risk, primarily by sustaining cancer stemness and immune-escaping capacities in residual tumor cells. In contrast, acute or therapy-induced inflammation may transiently suppress relapse through immune activation—though this protective effect remains subordinate to the overarching pro-tumorigenic trajectory (56). In summary, these inflammatory factors play pivotal roles in the development and metastasis of thyroid carcinoma, providing new perspectives and potential therapeutic targets for its treatment and prognosis.

The second cluster explores the link between inflammation and the diagnosis and management of thyroid carcinoma. In terms of diagnosis, inflammation primarily manifests through serum inflammatory biomarkers (e.g., NMPLR, LMR, NLR, PLR, SII) and their role in diagnosing and predicting different types of thyroid carcinoma. Given the differing treatment approaches for aDTC and ATC, rapid and reliable diagnosis of these conditions is crucial. While pathological examination via fine-needle aspiration or core needle biopsy is considered the gold standard for ATC diagnosis, it often requires significant time to obtain results. Recent studies have proposed the NMPLR, a composite index based on four immune cell types, as a biomarker for differentiating ATC from aDTC and predicting prognosis (63). Ahn et al. reported that a low LMR is associated with poor prognosis in ATC (64). Yokota et al. demonstrated that a preoperative low LMR strongly predicts high-risk recurrence in thyroid carcinoma patients (65). However, other studies suggest that a high NLR is a risk factor for thyroid carcinoma recurrence (36). Generally, elevated NLR, PLR, and SII are associated with poorer cancer outcomes. While NLR, PLR, and SII may serve as useful biomarkers indicating increased clinicopathological aggressiveness in MTC, their utility in independently predicting lymph node or distant metastasis appears limited (66). In 2023, Roberta Modica et al. evaluated the potential role of NLR, PLR, and SII as biomarkers in MTC. Although high NLR, PLR, and SII are linked to adverse outcomes in several cancer types, their role in MTC patients remains undetermined. Future research should focus on measuring these inflammatory markers during the disease course to determine their utility in early recurrence detection (67).In terms of treatment, inflammation in thyroid carcinoma is primarily studied in the context of postoperative infections. Surgery, as a therapeutic intervention, inevitably causes trauma to the patient, triggering an inflammatory response at the wound site and leading to transient increases in inflammatory factors such as TNF-α, Gal-3, and IL-6 (68). In thyroid carcinoma treatment, traditional open surgery and endoscopic surgery have been the primary options (69, 70). However, with the growing emphasis on postoperative quality of life in contemporary clinical practice, aesthetic outcomes in head and neck surgery have become a critical factor in enhancing patient satisfaction. Consequently, research has increasingly focused on endoscopic thyroidectomy, a minimally invasive approach valued for its aesthetic benefits (71). Beyond its cosmetic advantages, endoscopic thyroidectomy significantly reduces inflammatory responses at the incision site. Differences in postoperative inflammatory factor levels have been observed between traditional open surgery and endoscopic surgery. Due to its minimally invasive nature, endoscopic surgery effectively lowers the risk of postoperative bleeding, thereby reducing patient pain. In a 2021 study by Li He et al., measurements of TNF-α, Gal-3, and IL-6 levels before and after surgery in thyroid carcinoma patients revealed increased levels of these inflammatory factors on the first postoperative day in both traditional open surgery and endoscopic surgery groups. This indicates an acute inflammatory response post-surgery, but the increase was significantly lower in the endoscopic surgery group, demonstrating its effectiveness in reducing postoperative inflammation. Thus, endoscopic surgery not only minimizes postoperative pain but also mitigates inflammatory responses, significantly improving the quality of life for thyroid carcinoma patients (72).

The third cluster focuses on the tumor microenvironment (TME) of thyroid carcinoma. Tumors, as unique speciation events, employ an evolutionary strategy termed pathological niche construction by modulating microenvironmental conditions and resources to foster their own proliferation (73).The tumor microenvironment in thyroid carcinoma resembles a sophisticated ecosystem, comprising both malignant and non-malignant cellular components, stromal elements, vascular networks, and immune cell populations that collectively establish an intricate web of resource acquisition and metabolic exchange (74).Through dynamic reciprocal interactions, malignant cells undergo continuous co-evolutionary adaptation with neighboring tumor cells and stromal components, thereby augmenting their oncogenic fitness (75).The microenvironmental constituents collectively exert pivotal influence on thyroid cancer pathogenesis, mediating disease progression through multidimensional pathways encompassing immune escape strategies, extracellular matrix reorganization, and metabolic intervention. By studying immune cells within the TME of thyroid carcinoma, cancer-related inflammatory factors may serve as valuable targets for novel diagnostic and therapeutic strategies (76). During tumor development, interactions between the tumor and its surrounding immune microenvironment promote immune tolerance, leading to immune escape and ultimately preventing the complete eradication of the tumor. Studies have shown that M2 macrophages, Tregs, monocytes, neutrophils, dendritic cells (DCs), mast cells (MCs), and M0 macrophages within the TME contribute to tumor promotion in papillary thyroid carcinoma (PTC). In contrast, M1 macrophages, CD8+ T cells, B cells, NK cells, and T follicular helper (TFH) cells (with weaker evidence for eosinophils, γδ T cells, and Th17 cells) exert anti-tumor effects. During PTC progression, overall immune activity increases, with significant elevations in the abundance and proportions of pro-tumor immune cells (M2 macrophages, Tregs, monocytes, neutrophils, DCs, MCs, and M0 macrophages) (33).Additionally, most anaplastic thyroid carcinoma (ATC) tumor cells are PD-L1 positive, and their TME is enriched with PD-1/PD-L1-positive infiltrating lymphocytes. Current research on immunotherapy for ATC patients indicates that ATC tumors express immune markers such as PD-L1, which has become a recent hotspot in inflammation and thyroid carcinoma research. The American Thyroid Association guidelines recommend checkpoint inhibitors (e.g., PD-L1, PD-1) as first-line or advanced therapy for stage IVC ATC patients with high PD-L1 expression in the absence of other targetable alterations (77). Cancer immunotherapy may represent an alternative treatment for tumors unresponsive to conventional therapies, such as recurrent or persistent differentiated thyroid carcinoma (DTC), poorly differentiated thyroid carcinoma (PDTC), or ATC. Monoclonal antibodies targeting PD-1/PD-L1, such as pembrolizumab, are currently available (76). Cancer-associated fibroblasts (CAFs), fundamental cellular architects of the tumor microenvironment, actively remodel the extracellular matrix (ECM) through elevated secretion of structural proteins such as fibronectin and type I collagen, while preferentially expressing the pathologically significant oncofetal fibronectin isoform (78).Cancer-associated fibroblasts (CAFs) orchestrate tumorigenesis through paracrine secretion of cytokines and growth factors that coordinately regulate cancer cell proliferation, survival, and stemness, while driving intratumoral angiogenesis and modulating immune cell recruitment and differentiation (74).Furthermore, CAFs mediate stromal ECM remodeling by upregulating the expression and activation of matrix metalloproteinases (MMPs). This CAF-driven matrix reorganization potently facilitates cancer cell migration and invasion (79). Metabolically, this intervention manifests through lactate accumulation and microenvironmental acidification - a phenomenon termed “nutrient competition.” Tumor cells predominantly generate lactate via the Warburg effect (aerobic glycolysis), consequently inducing acidification of the tumor microenvironment (TME) (80). As tumor proliferation advances, its expansive growth frequently outpaces vascular supply capacity, thereby generating a pathological microenvironment characterized by regional hypoxia, nutrient deprivation, and accumulated acidic metabolites. This dysregulated micro-ecosystem stands in stark contrast to the metabolically orchestrated milieu observed in healthy tissues (81, 82). The necrotic cellular debris interacts synergistically with tumor-derived proinflammatory factors, collectively forging a hostile ‘toxic swamp’-like microenvironment. This distinctive ‘tumor swamp’ ecology not only furnishes malignant cells with unique survival advantages but, more critically, imposes formidable selective pressures that ultimately drive the evolution of adaptive tumor clones with enhanced migratory and metastatic competence (75, 83).

The fourth cluster investigates the association between Hashimoto’s thyroiditis (HT) and thyroid carcinoma. Hashimoto’s thyroiditis is the most common inflammatory thyroid disorder and the leading cause of hypothyroidism. The relationship between HT and papillary thyroid carcinoma (PTC) was first described by Dailey et al. in 1955 (84). Since its initial proposal, the association between these conditions has been extensively studied and remains highly debated (85). Some scholars argue that HT may promote the development of PTC, while others suggest that HT could act as a protective factor against PTC (86). In a 2024 meta-analysis by Yali Le et al., the study aimed to examine the risk relationship between Hashimoto’s thyroiditis (HT) and thyroid carcinoma (TC), with a focus on gender differences. By conducting a comprehensive search across online databases, including PubMed, Cochrane Library, EMBASE, and Web of Science, the study analyzed the correlation between HT and TC. The results indicated a significant association between HT and TC (OR: 2.22, 95% CI: 1.85–2.67), suggesting that HT may increase the risk of TC. Additionally, the study performed a supplementary analysis of gender-specific data to determine the odds ratios (ORs) for females and males, as well as the prevalence of TC in HT patients by gender. However, the supplementary analysis revealed no statistically significant difference in TC prevalence between female (0.31, 95% CI: 0.17–0.45) and male (0.37, 95% CI: 0.21–0.53) HT patients. The final discussion highlighted that HT patients have a higher incidence of TC compared to non-HT patients. HT is considered a precancerous lesion for TC, and autoimmune thyroiditis may be a risk factor for TC, with the inflammatory processes of HT playing a significant role in TC development (50). The malignant transformation of the thyroid may be driven by cellular mediators from immune cells in a chronic inflammatory state or by elevated TSH levels stimulating follicular epithelial cell proliferation (87). Evidence suggests that HT patients have a higher risk of thyroid carcinoma compared to the general population aged 9–15. According to relevant literature, the pathogenesis of HT-associated thyroid carcinoma may involve apoptosis of thyroid epithelial cells, diffuse lymphocyte infiltration, fibrous replacement, and follicular destruction in HT. Therefore, evaluating the association between autoimmune thyroiditis and PTC is meaningful (88). Furthermore, since oxidative stress (OS) and inflammatory environments are implicated in the development of various cancers, studies have further explored the systemic and microenvironmental involvement of oxidative stress and inflammatory markers in HT and PTC patients. The results showed increased systemic OS in thyroid disease patients, with PTC-only patients exhibiting higher systemic OS levels than those with both PTC and HT, potentially leading to worse prognoses in PTC-only patients compared to those with PTC and HT (89).

The fifth cluster explores the link between obesity and thyroid carcinoma risk. Over recent decades, the global prevalence of overweight and obesity has surged, with the incidence of differentiated thyroid carcinoma (DTC) increasing alongside rising obesity rates, supporting a potential correlation between the two conditions (90). Obesity is defined as a chronic, low-grade inflammatory and non-communicable disease, often accompanied by a state of mild chronic inflammation characterized by elevated systemic inflammatory markers and nonspecific activation of the immune system (91). Adipose tissue is not merely a site for energy storage; it should also be regarded as an endocrine organ composed of various cell types, including adipocytes, preadipocytes, and immune cells. Adipocytes readily secrete pro-inflammatory adipokines, such as tumor necrosis factor-α (TNF-α), monocyte chemoattractant protein-1 (MCP-1), and interleukin-6 (IL-6), which recruit immune cells and amplify inflammatory processes, thereby promoting cancer development, progression, and metastasis (92). It is now widely accepted that obesity-associated immune dysregulation, compensatory hyperinsulinemia, hyperlipidemia, and enhanced oxidative stress are risk factors for cancer development and/or progression (93). Some data suggest that increased inflammasome activity in adipose tissue is a key mediator of obesity-induced inflammation and insulin resistance (94). Inflammasomes can be activated by fatty acids and high glucose levels, linking metabolic danger signals to the activation of inflammation and thyroid carcinoma development. Thus, inflammasome activation may represent a critical step in obesity-related thyroid carcinoma pathogenesis (95).Additionally, obesity influences the secretion of adipokines (hormones secreted by adipocytes), with data supporting the notion that adipokines such as leptin and adiponectin may be directly related to adiposity. Increased leptin and decreased adiponectin levels are well-documented examples (96). Scientific research has robustly demonstrated an inverse correlation between adiponectin (APN) levels and the risk of endocrine tumors (97). Given that obesity often leads to hypoadiponectinemia, the increased risk of thyroid carcinoma in obese individuals may be at least partially attributed to the loss of this hormone-induced immunosuppressive effect (91). Furthermore, high-fat diet (HFD)-induced obesity accelerates thyroid carcinoma growth and progression, particularly by shortening survival and promoting anaplastic transformation. The core mechanism underlying these changes lies in HFD-induced elevation of leptin levels, which activates the JAK2-STAT3 signaling pathway. Preclinical studies using the STAT3-specific inhibitor S3I-201 have further confirmed the pivotal role of leptin downstream effectors in obesity-related thyroid carcinoma development (98).In summary, obesity promotes the initiation and progression of thyroid carcinoma through multiple mechanisms, including chronic inflammation, adipokine imbalance, HFD-induced metabolic dysregulation, and inflammasome activation. These mechanisms provide potential targets for the prevention and treatment of thyroid carcinoma in the future.

Using CiteSpace for keyword co-occurrence analysis, this study highlights the field’s trending topics, revealing emerging frontiers and shifts in research hotspots over time. Future research will increasingly focus on the ‘systemic immune-inflammation index’ and ‘Th17 cells,’ reflecting their growing significance in the study of inflammation and thyroid carcinoma.

The systemic immune-inflammation index (SII) is a novel inflammatory marker derived from the absolute counts of neutrophils, lymphocytes, and platelets, which can be calculated from routine complete blood count (CBC) data. In thyroid carcinoma research, this inflammatory marker is manifested in four key aspects: 1)Association with Tumor Multifocality: SII is significantly higher in patients with differentiated thyroid carcinoma (DTC) compared to healthy individuals, and elevated SII values are strongly correlated with tumor multifocality (i.e., the presence of multiple independent tumor foci) (p=0.01). This suggests that SII may reflect the aggressive biological behavior of tumors, although it shows no significant association with tumor diameter, histological type, or neural/lymphatic/capsular invasion (99). 2)Predictive Marker for Lymph Node Metastasis: In thyroid carcinoma patients, the diagnosis of central lymph node metastasis (CLNM) is complicated by false negatives in imaging studies, leading to many clinically node-negative (cN0) patients having undetected central lymph node metastases. This can interfere with clinical decision-making. Fortunately, a retrospective study of cN0 papillary thyroid carcinoma (PTC) patients undergoing radical surgery determined CLNM through postoperative histopathological examination. A comprehensive analysis of various inflammatory biomarkers concluded that SII effectively predicts CLNM in cN0 PTC and papillary thyroid microcarcinoma (PTMC) patients. This finding is significant for distinguishing between non-CLNM and CLNM PTC patients, as it may help clinicians avoid unnecessary prophylactic central lymph node dissection in patients without actual lymph node metastasis (100). Additionally, preoperative SII is an independent risk factor for lateral lymph node metastasis (LLNM). Multivariate analysis shows that elevated SII is associated with an increased risk of LLNM (101). 3)Potential Link to Thyroid Carcinoma Prognosis: SII is believed to be associated with thyroid carcinoma prognosis. Studies indicate that higher SII values may correlate with aggressive tumor behavior and poor prognosis, although its precise prognostic value requires validation through long-term follow-up studies. For example, high SII may indirectly influence metastasis risk or treatment response by promoting inflammatory reactions in the tumor microenvironment (102).4)Differentiation Between Benign and Malignant Thyroid Diseases: With the rising incidence of thyroid carcinoma, the healthcare system faces increased pressure and a higher risk of overtreatment. In this context, accurately distinguishing between thyroid carcinoma and benign nodules preoperatively is crucial. This step is essential to avoid unnecessary surgical interventions, reduce patient burden, minimize complication risks, and implement more precise treatment strategies (103). In a 2023 study by Selahattin Vural et al., preoperative SII levels were significantly higher in PTC patients compared to those with benign thyroid diseases, suggesting that this simple marker can differentiate between benign and malignant thyroid conditions. Moreover, high SII is associated with increased invasiveness in PTC (104).

The role of SII in thyroid carcinoma has become a research hotspot in recent years, and future studies will delve deeper into its mechanisms and applications.

Helper T cells 17 (Th17) are a recently identified subset of T cells capable of secreting interleukin-17 (IL-17), playing a significant role in autoimmune diseases and host defense mechanisms. Chronic inflammation and carcinogenesis are often associated with the relative roles of two specific lymphocyte subsets: regulatory T cells (Tregs) and Th17 cells (105). The pro-inflammatory IL-23/Th17 axis has emerged as a critical mediator in inflammation-related cancers. The role of the Th17 spectrum, including immune cells, cytokines, chemokines, and their receptors, may either be protective or act as a trigger for thyroid tumors, depending on the histological classification of the tumor (32). Research by Denise Faria Galano Carvalho et al. demonstrated an association between Th17 cell infiltration and poor prognosis, suggesting that Th17 cells may suppress anti-tumor responses (106). Given that IL-17, expressed by Th17 cells, is involved in the pathogenesis of chronic inflammatory responses and is known to induce the production of high concentrations of IL-1β, TNF-α, TGF-β, CC chemokine ligand 2 (CCL2), and matrix metalloproteinase mediators—all of which are prevalent in the tumor microenvironment—this cytokine represents a potential therapeutic target in thyroid carcinoma (107). In 2023, Sohini Banerjee et al. found that IL-17A expression is significantly correlated with smaller thyroid tumor diameter and disease progression, highlighting its importance as a biomarker for early diagnosis and future prognosis in papillary thyroid carcinoma (PTC) (108).

## 5 Limitations of the study

This study has several methodological limitations that require explicit acknowledgment. First, regarding data sources, we deliberately restricted our literature search to the Web of Science (WoS) database, excluding other repositories such as PubMed and Scopus. This decision was based on two primary considerations (1): WoS offers unique advantages in interdisciplinary coverage, enabling more comprehensive cross-disciplinary analysis compared to PubMed’s specialized focus on biomedical and life sciences; and (2) WoS provides unparalleled citation tracking capabilities (both forward and backward citations), allowing for robust assessment of academic impact through complete citation network analysis - a distinctive feature that sets it apart from other databases. The WoS platform integrates sophisticated bibliometric tools that facilitate high-precision data mining, including citation frequency analysis and journal impact factor evaluation. These advanced features enable precise identification of research hotspots and emerging frontiers, offering analytical depth unavailable in general-purpose databases. Nevertheless, we recognize that this selective approach may introduce certain biases, particularly the potential omission of significant biomedical literature not indexed in WoS. An additional limitation stems from our inclusion of English-language publications only, which may overlook important contributions published in other languages, especially from high-output non-English-speaking countries. However, we note that English remains the predominant language of scientific communication, with most prolific researchers publishing in English regardless of their native language. Furthermore, our 24-year timeframe, while ensuring focus on contemporary trends, means that recently published works may not yet have accumulated substantial citation counts. It is important to emphasize that these limitations represent inherent challenges in bibliometric research methodology. We have carefully considered their potential impact throughout our study design and results interpretation to ensure balanced and meaningful conclusions.

## 6 Conclusion

In summary, this study employs bibliometric methods to conduct an in-depth evaluation and visual presentation of the literature corpus in the field of inflammation and thyroid carcinoma. Using the Web of Science Core Collection (WoSCC) as the data source, we meticulously examined English articles and reviews published between January 1, 2000, and November 12, 2024. Leveraging bibliometric tools such as CiteSpace and VOSviewer, our analysis revealed the current research landscape, outlined core themes, and predicted emerging trends in inflammation-related thyroid carcinoma research. Key research focuses include the tumor microenvironment, the expression of inflammation-related factors, the role of inflammation in thyroid carcinoma management, and the link between Hashimoto’s thyroiditis and thyroid carcinoma. This comprehensive bibliometric analysis not only highlights the achievements of existing research but also lays a solid foundation for future academic exploration and progress in understanding the relationship between inflammation and thyroid carcinoma. Through this systematic approach, we provide researchers and practitioners in the field with a valuable roadmap and research directions for future studies.

## Data Availability

The original contributions presented in the study are included in the article/supplementary material. Further inquiries can be directed to the corresponding author.
